# A 17-Gene Signature Predicted Prognosis in Renal Cell Carcinoma

**DOI:** 10.1155/2020/8352809

**Published:** 2020-02-26

**Authors:** Fan Li, Weifeng Hu, Wei Zhang, Guohao Li, Yonglian Guo

**Affiliations:** Department of Urology, The Central Hospital of Wuhan, Tongji Medical College, Huazhong University of Science and Technology, Wuhan 430014, China

## Abstract

Renal cell carcinoma (RCC), which was one of the most common malignant tumors in urinary system, had gradually increased incidence and mortality in recent years. Although significant advances had been made in molecular and biology research on the pathogenesis of RCC, effective treatments and prognostic indicators were still lacking. In order to predict the prognosis of RCC better, we identified 17 genes that were associated with the overall survival (OS) of RCC patients from The Cancer Genome Atlas (TCGA) dataset and a 17-gene signature was developed. Through SurvExpress, we analyzed the expression differences of the 17 genes and their correlation with the survival of RCC patients in five datasets (ZHAO, TCGA, KIPAN, KIRC, and KIRP), and then evaluated the survival prognostic significance of the 17-gene signature for RCC. Our results showed that the 17-gene signature had a predictive prognostic value not only in single pathologic RCC, but also in multiple pathologic types of RCC. In conclusion, the 17-gene signature model was related to the survival of RCC patients and could help predict the prognosis with significant clinical implications.

## 1. Introduction

Renal cell carcinoma (RCC), the main form of kidney cancer, was the second most common cancer in the urological system and accounted for approximately 3% of malignant neoplasms worldwide [1]. It included a variety of malignant tumors that originated from renal tubular epithelial cells and up to 85% of kidney cancer belonged to RCC [2]. The incidence and mortality had gradually increased in recent years [3]. RCC was usually sporadic (about 96%), but occasionally familial (about 4%), and it was often associated with specific gene mutations [4]. It occurred more frequently in men than in women (ratio of 1.7 : 1), and most people were older, with an average age of 64 years [5, 6]. Clear cell renal cell carcinoma (ccRCC) accounted for 80-90% of RCC [7, 8] and was the predominant histological subtype characterized by its resistance to conventional chemotherapy and radiotherapy [9].

Although surgical tumor resection was an effective treatment for RCC at present [10–12], radical surgery cannot completely cure RCC [13, 14]. Recent studies had focused on the possibility of combining strategy for improving the therapeutic value of existing standard therapies, including chemotherapy and radiotherapy [15, 16]; however, RCC was not sensitive to radiotherapy and chemotherapy [17, 18]. Patients in early stage of RCC (>50% of cases) had a favorable prognosis after nephrectomy, with the 5-year survival rate of about 81%. However, 10-30% of patients with early RCC would undergo tumor recurrence after nephrectomy [19], and about 20% of patients had presented metastatic diseases when they came for treatment [20]. The common metastatic sites of RCC included the brain, lung, and bones. In patients with metastatic diseases, the median survival time ranged from 6 to 12 months [21]. For advanced stage of RCC, systemic therapy was the foundational treatment. On account of the extremely high rate of local invasion and metastasis, as well as the resistance to chemotherapy and radiotherapy, over 30% of RCC patients with metastasis had a 5-year survival rate below 20% [22, 23], which indicated a poor long-term prognosis. Therefore, it was essential to research on the development mechanism of RCC at molecular level. This may help determine the invasion and metastasis ability of RCC and its malignancy, predict the prognosis of RCC, develop a reasonable treatment program, and provide new ideas for molecular targeted therapy.

In order to solve this unmet need, a prognostic 17-gene signature model was developed. We hypothesized that this 17-gene signature may reflect the risk level of adverse clinical outcomes in RCC, which may be useful to correctly predict the overall survival (OS) of RCC and aid the clinicians in treatment planning.

## 2. Materials and Methods

### 2.1. Selection and Analysis of Databases

**Figure 1:**
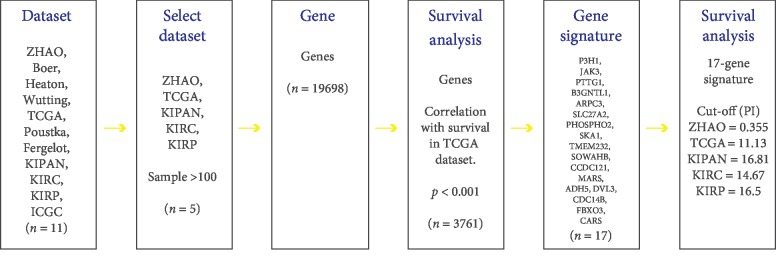
The procedure flow chart of SurvExpress. Schematic overview of the procedure used in our study to construct the 17-gene signature.

All the data were analyzed using SurvExpress (http://bioinformatica.mty.itesm.mx:8080/Biomatec/SurvivaX.jsp). SurvExpress was a comprehensive gene expression analysis tool that was based on a number of databases. It can provide risk assessment and survival analysis in many cancer datasets [24, 25]. In this analysis, SurvExpress was used to provide Kaplan-Meier log rank analysis, risk evaluation, etc. For RCC, SurvExpress incorporated 11 independent public databases. In our study, we only included databases with a sample number greater than 100, and five databases (ZHAO, TCGA, KIPAN, KIRC and KIRP) were screened out, containing a total of 19698 coding genes. Then, 3761 genes associated with the prognosis of RCC were selected out in the TCGA database from the 19698 genes (*p* < 0.001). After that, the 3761 genes were sorted by *p* value and the top 99 genes were combined. In this combination, genes with a significant difference in expression levels were picked out to form a 17-gene signature (Figure 1). Their gene ID, full name, and function were shown in Table 1.

### 2.2. Study Design

**Figure 2:**
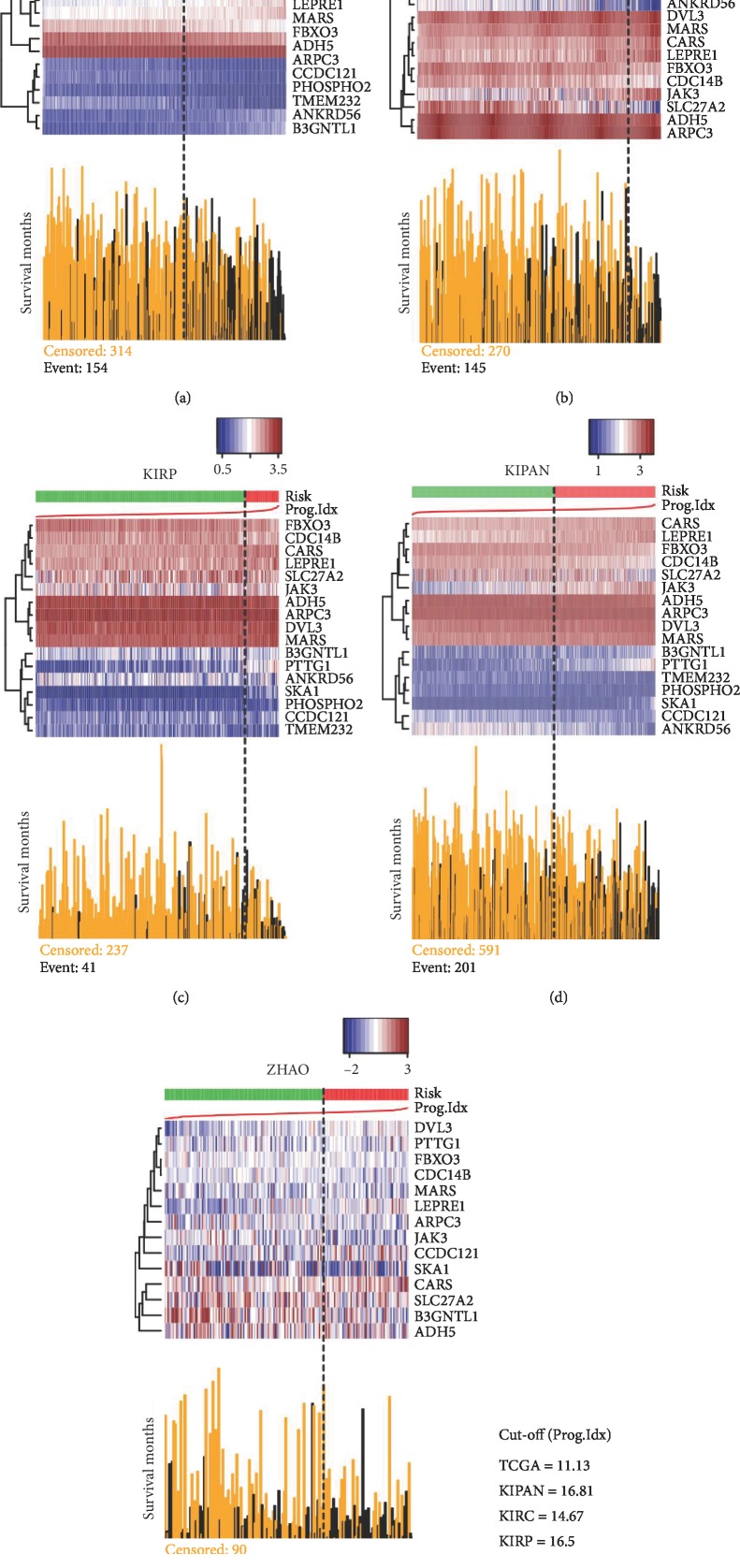
Sort PI from low to high and select the optimal cut-off value. Then, the patients were divided into high-risk and low-risk groups according to the optimal cut-off value. Red represented high-risk group and green represented low-risk group. Each figure was composed of the upper gene expression heat map and the nether prognostic index graph. (a) TCGA database contained 468 patients (*n* = 154 in the high-risk group, *n* = 314 in the low-risk group) and the optimal cut-off value of PI was 11.13. (b) KIRC database contained 415 patients (*n* = 145 in the high-risk group, *n* = 270 in the low-risk group) and the optimal cut-off value of PI was 14.67. (c) KIRP database contained 278 patients (*n* = 41 in the high-risk group, *n* = 237 in the low-risk group) and the optimal cut-off value of PI was 16.5. (d) KIPAN database contained 792 patients (*n* = 201 in the high-risk group, *n* = 591 in the low-risk group) and the optimal cut-off value of PI was 16.81. (e) ZHAO database contained 177 patients (*n* = 87 in the high-risk group, *n* = 90 in the low-risk group) and the optimal cut-off value of PI was 0.355. (f) The optimal cut-off value of the five databases (TCGA: 11.13; KIPAN: 16.81; KIRC: 14.67; KIRP: 16.5; ZHAO: 0.355).

Using SurvExpress, we analyzed the expression differences of the 17 genes and their correlation with the survival of RCC patients in the five datasets (ZHAO, TCGA, KIPAN, KIRC and KIRP), and then evaluated the survival prognostic significance of the 17-gene signature for RCC (Table 1). The prognostic index (PI), which was also known as the risk score, was often used to perform risk assessment and generate risk groups. The PI was the linear component of the Cox model, PI = b1x1 + b2x2+⋯+bixi, where the bi can be obtained from the Cox fitting and xi was the expression value. Each bi can be construed as a risk parameter [26]. According to the PI formula, each patient had a corresponding PI. Sort PI from low to high and select the optimal cut-off value. Then, the patients were divided into high-risk and low-risk groups according to the optimal cut-off value (Figure 2).

### 2.3. Statistical Analysis

To evaluate the prognostic value of the 17-gene signature, Kaplan-Meier estimator was used to plot survival curves and the log-rank test was performed to compare the differences between the two groups [26, 27]. Kaplan-Meier can also be used to provide the receiver operating characteristic (ROC) curve to determine the accuracy of the 17-gene signature in predicting the prognosis of RCC. The ROC analysis was a tool used to describe the discrimination accuracy of a diagnostic test or prediction model [28]. One of the most commonly used ROC summary indices was the area under the ROC curve (AUC). The AUC values were calculated from the ROC curve [28, 29]. *p* < 0.05 was considered to be statistically significant.

## 3. Results

### 3.1. Survival Analysis of the 17 Genes in TCGA Dataset

We analyzed the expression differences of the 17 genes in TCGA dataset with SurvExpress. The gene ID, full name, and function were obtained from the NCBI FTP site and GeneCards. Our analysis showed that the 17 genes all had significant prognostic differences in the TCGA database (*p* < 0.001) (Table 1).

### 3.2. Sort PI from Low to High and Select the Optimal Cut-off Value

Sort PI from low to high and select the optimal cut-off value. The optimal cut-off values of PI in the five databases (TCGA, KIRC, KIRP, KIPAN, and ZHAO) were 11.13, 14.67, 16.5, 16.81, and 0.355 (Figure 1). Then, the patients were divided into high-risk and low-risk groups according to the optimal cut-off value (Figure 2).

### 3.3. The 17-Gene Signature Showed a Predictive Prognostic Significance in RCC

**Figure 3:**
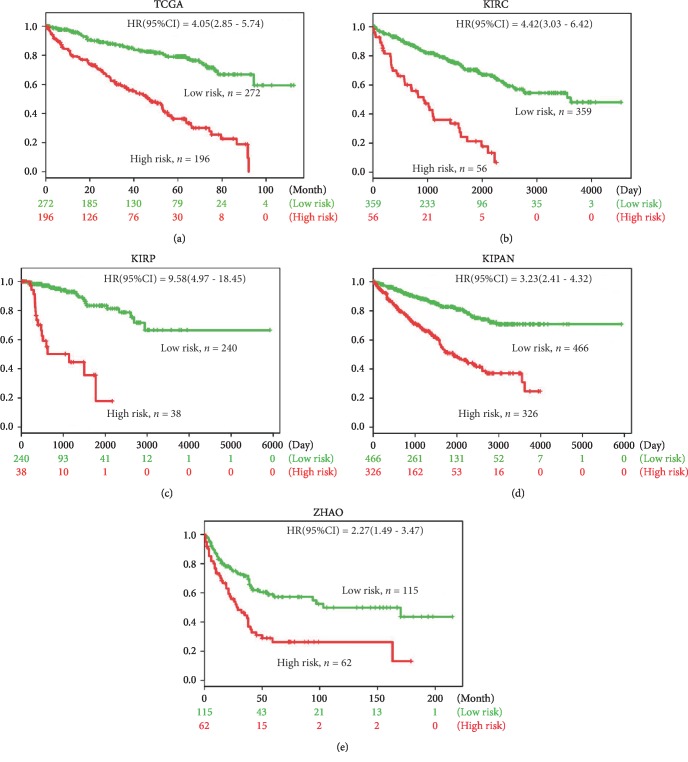
Survival differences between the predicted low-risk and high-risk groups were evaluated through Kaplan-Meier survival curves. The results indicated that the prognosis of patients with low risk was all significantly better than that in the high-risk group in the TCGA (a), KIRC (b), KIRP (c), KIPAN (d), and ZHAO (e) databases (*p* < 0.001).

Survival differences between predicted low-risk and high-risk groups were evaluated with Kaplan-Meier survival curves (Figure 3). TCGA data showed that the prognosis of patients with low risk (*n* = 272) was significantly better than high-risk group patients (*n* = 196) (HR (95%CI) = 4.05 (2.85-5.74), *p* < 0.001) (Figure 3(a)). KIRC database was a corresponding database for kidney renal clear cell carcinoma, which also showed a prognostic value of the 17-gene signature (*p* < 0.001) (Figure 3(b)). The research object of KIRP database was kidney renal papillary cell carcinoma. Likewise, our analysis found a significant prognostic difference between the two groups (*n* = 240 in the low-risk group and *n* = 38 in the high-risk group) (HR (95%CI) = 9.58 (4.97-18.45), *p* < 0.001) (Figure 3(c)). KIPAN incorporated three types of kidney cancer, including kidney chromophobe, kidney renal clear cell carcinoma, and kidney renal papillary cell carcinoma. For the comprehensive KIPAN database, our 17-gene signature had a significant predictive significance as well (*p* < 0.001) (Figure 3(d)). ZHAO database (GSE3538) revealed a significant prognostic difference between the low-risk group (*n* = 115) and the high-risk group (*n* = 62) (HR (95%CI) = 2.27 (1.49-3.47), *p* < 0.001), indicating that the 17-gene signature can also be used as a prognostic indicator in the ZHAO database (Figure 3(e)). In conclusion, our results demonstrated that the 17-gene signature had a predictive prognostic value not only in single pathologic RCC but also in multiple pathologic types of RCC.

### 3.4. The Accuracy Analysis of the 17-Gene Signature in Predicting the Prognosis of RCC

**Figure 4:**
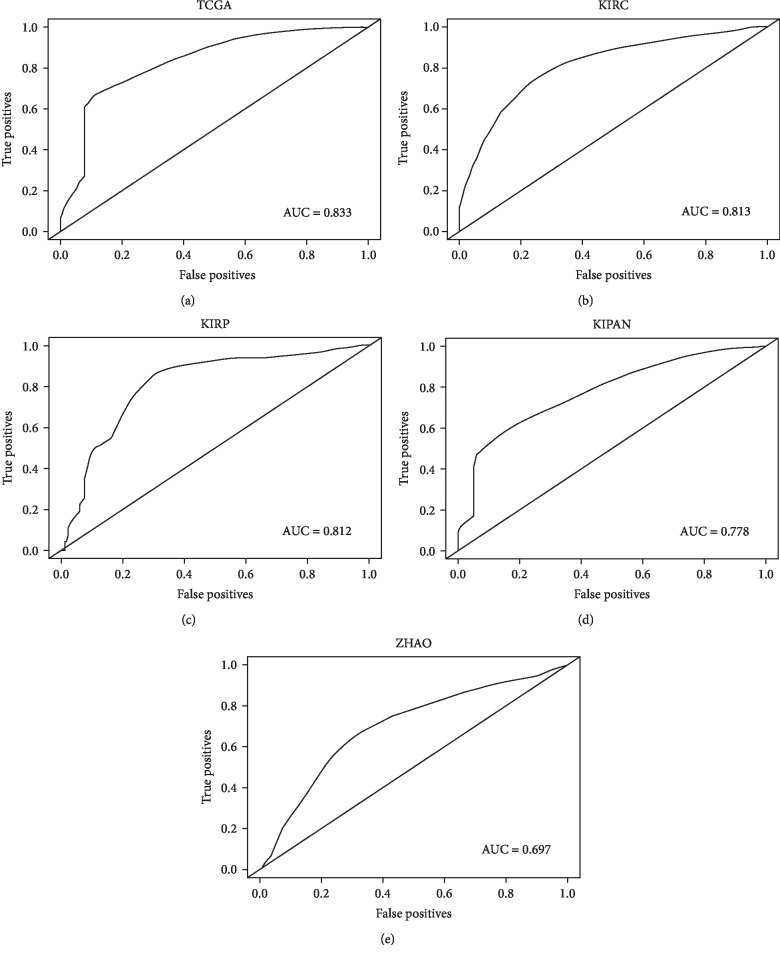
The accuracy analysis of the 17-gene signature in predicting the prognosis of RCC. Kaplan-Meier was used to obtain ROC curves. The results showed that the ROC curves all had an area under the ROC curve (AUC) of greater than 0.6 in the five databases (TCGA, KIRC, KIRP, KIPAN, and ZHAO) (*p* < 0.001).

In order to determine the accuracy of the 17-gene signature in predicting the prognosis of RCC, we conducted the true positives and false positives analysis by SurvExpress (Figure 4). The receiver operating characteristic curve (ROC) for predicting 5-year survival was obtained according to PI. The time points provided in Figures 4(a) and 4(b) were months, while the time points in Figures 4(c)–4(e) were days. Our analysis revealed that in different time points, different pathological types of RCC, and different databases, the ROC curves all had an area under the ROC curve (AUC) of greater than 0.6 (TCGA: 0.833, KIRC: 0.813, KIRP: 0.812, KIPAN: 0.778, and ZHAO: 0.697), suggesting that the 17-gene signature had certain accuracy in predicting RCC prognosis.

## 4. Discussion

RCC was the third most common malignancy in the urogenital system, which represented about 2% to 3% of cancers in adults [30]. The genesis and progression of RCC involved various factors, including carcinogenic substances and environmental factors [31, 32]. Smoking and obesity were considered to be risk factors for the development of RCC [33]. RCC was divided into four histological main subtypes [34]. In general, WHO distinguished RCC into clear cell RCC (ccRCC) and nonccRCC. Thereinto, ccRCC was the predominant subtype of RCC [35].

RCC was classified as an “immunogenic” tumor based on the following characteristics: spontaneous regression of the tumor, high levels of T cell infiltration in the tumor, and reactivity to immunotherapy such as interleukin-2 (IL-2) and interferon alpha (IFN-*α*) [36]. However, due to low efficacy and high adverse reactions, these therapeutic measures were not ideal. 30% of patients had already presented advanced disease or other metastatic diseases when they came for treatment [37]. Eventually, about 40% of patients died of metastases [38]. Therefore, it was urgently needed to find potential prognostic biomarkers to predict the prognosis of RCC and draw up rational treatment programs.

In the present study, we first identified 17 genes associated with OS of RCC patients from the TCGA dataset. Through SurvExpress, we analyzed the expression differences of the 17 genes in RCC patients in the TCGA dataset. Our analysis showed that the 17 genes had significant prognostic differences in the TCGA database (*p* < 0.001). Then, a 17-gene signature model was developed and its correlation with the survival of RCC patients in the five datasets was analyzed, respectively. The patients were divided into low-risk and high-risk groups according to the optimal cut-off value. Survival differences between the predicted low-risk and high-risk groups were evaluated with Kaplan-Meier survival curves. The results presented that the prognosis of patients with low risk was all significantly better than that of high-risk group patients in the five databases (*p* < 0.001), suggesting that the 17-gene signature had a predictive prognostic value not only in single pathologic RCC but also in multiple pathologic types of RCC.

Finally, in order to determine the accuracy of the 17-gene signature in predicting the prognosis of RCC, the receiver operating characteristic curve (ROC) for predicting 5-year survival was obtained according to PI. The value of AUC was the size of the area under the ROC curve. Typically, the AUC value was between 0.5 and 1.0, and larger AUC represented better performance [30, 31]. Our analysis revealed that the ROC curves all had an AUC of greater than 0.6 in the five databases (*p* < 0.001), suggesting that the 17-gene signature had certain accuracy in predicting the prognosis of RCC.

## 5. Conclusions

In summary, our study demonstrated a survival prognostic significance of a 17-gene signature for RCC. This may be a potential prognostic tool to improve the adverse clinical outcomes of RCC patients currently. Further prospective studies were needed to determine whether the 17-gene signature can be used clinically to benefit RCC patients.

## Figures and Tables

**Table 1:** The expression differences of the 17 genes in TCGA dataset with SurvExpress.

Gene	Gene ID	Full name	Function	HR (95% CI)	*p* value
P3H1	64175	Prolyl 3-hydroxylase 1	Has prolyl 3-hydroxylase activity and growth suppressive activity in fibroblasts and involve in the secretory pathway of cells	2.96 (2.09-4.18)	9.25*E*-10
JAK3	3718	Janus kinase 3	Nonreceptor tyrosine kinase involved in various processes such as cell growth, development, or differentiation. Mediate essential signaling in both innate and adaptive immunity and hematopoiesis during T cell development.	3.04 (2.13-4.33)	8.521*E*-10
PTTG1	9232	Pituitary tumor-transforming 1	Regulatory protein which plays a central role in chromosome stability, the p53/TP53 pathway, and DNA repair	3.07 (2.15-4.4)	7.852*E*-10
B3GNTL1	146712	BetaGal beta-1,3-N-acetylglucosaminyltransferase like 1	Putative glycosyltransferase.	3.02 (2.12-4.29)	7.533*E*-10
ARPC3	10094	Actin-related protein 2/3 complex subunit 3	Localize to the lamellipodia of stationary and locomote fibroblasts, inducing actin polymerization and potentially participating in lamellipodial protrusion	3.08 (2.15-4.41)	7*E*-10
SLC27A2	26458	Solute carrier family 27 (fatty acid transporter), member 2	Acyl-CoA synthetase probably involves in bile acid metabolism. May have additional roles in fatty acid metabolism and be involved in translocation of long-chain fatty acids across membranes	3.01 (2.12-4.28)	6.999*E*-10
PHOSPHO2	73373	Phosphatase, orphan 2	High activity toward pyridoxal 5′-phosphate. Also active at a much lower level toward phosphoethanolamine, pyrophosphate, phosphocholine, phospho-l-tyrosine, fructose-6-phosphate, p-nitrophenyl phosphate, and h-glycerophosphate.	3.01 (2.12-4.27)	6.661*E*-10
SKA1	220134	Spindle- and kinetochore-associated complex subunit 1	Component of the SKA1 complex that is essential for proper chromosome segregation. High expression levels of SKA1 were significantly associated with low DFS.	3.09 (2.16-4.41)	5.455*E*-10
TMEM232	642987	Transmembrane protein 232	Unclear.	3.00 (2.12-4.23)	4.299*E*-10
SOWAHB	345079	Sosondowah ankyrin repeat domain family member B	Unclear.	3.10 (2.17-4.42)	4.173*E*-10
CCDC121	403180	Coiled-coil domain-containing 121	Unclear.	3.14 (2-4.47)	2.512*E*-10
MARS	4141	Methionyl-tRNA synthetase	A protein-coding gene plays a role in the synthesis of ribosomal RNA in the nucleolus.	3.09 (2.18-4.37)	1.969*E*-10
ADH5	128	Alcohol dehydrogenase 5 (class III), chi polypeptide	Catalyze the oxidation of long-chain primary alcohols and the oxidation of S-(hydroxymethyl) glutathione. Also oxidize long-chain omega-hydroxy fatty acids.	3.19 (2.24-4.56)	1.649*E*-10
DVL3	1857	Dishevelled segment polarity protein 3	Involve in the signal transduction pathway mediated by multiple Wnt genes.	3.17 (2.23-4.49)	9.754*E*-11
CDC14B	8555	Cell division cycle 14B	Dual-specificity phosphatase involved in DNA damage response. Essential regulator of the G2 DNA damage checkpoint, a key activator of the anaphase-promoting complex/cyclosome (APC/C).	3.32 (2.32-4.74)	5.069*E*-11
FBXO3	26273	F-box protein 3	Substrate recognition component of the SCF (SKP1-CUL1-F-box protein)-type E3 ubiquitin ligase complex. Mediate the ubiquitination of HIPK2 and probably that of EP300, leading to rapid degradation by the proteasome.	3.32 (2.32-4.74)	4.352*E*-11
CARS	833	Cysteinyl-tRNA synthetase	An important tumor-suppressor gene. Alterations in this region is associated with Beckwith-Wiedemann syndrome; Wilms tumor; adrenocortical carcinoma; and lung, ovarian, and breast cancers, rhabdomyosarcoma.	3.69 (2.56-5.33)	2.533*E*-12

## Data Availability

The data used to support the findings of this study are included within the article.
